# Study on the Rheological Properties of BGAP Adhesive and Its Propellant

**DOI:** 10.3390/molecules30091967

**Published:** 2025-04-29

**Authors:** Yubao Shao, Siyu Xu, Huixiang Xu, Wuxi Xie, Zihao Zhang, Ergang Yao, Hanyu Jiang

**Affiliations:** Xi’an Modern Chemistry Research Institute, Xi’an 710065, China; 18040119707@163.com (Y.S.); xhx204@163.com (H.X.); xiewuxi@163.com (W.X.); zzh565399203@stu.xjtu.edu.cn (Z.Z.); yaoerg@126.com (E.Y.); jianghanyu612@126.com (H.J.)

**Keywords:** branched polyazide glycidyl ether (BGAP), chemical rheology method, apparent viscosity, curing, Al powder

## Abstract

In order to study the curing process of branched polyazide glycidyl ether (BGAP) binder and its propellant slurry at 50 to 70 °C, the rheological properties of BGAP binder and its propellant slurry were studied by chemical rheology. The results show that the viscosity coefficient of the uncured BGAP decreases gradually when the temperature increases, and when the plasticization ratio is 1.1, the viscosity coefficient of BGAP decreases first and then remains unchanged. After adding the curing agent, the chemical rheology method can be used to calculate whether the BGAP curing system still conforms to the power-law equation in a short time. The kinetic equation of the curing reaction, expressed by apparent viscosity, is deduced from the double Arrhenius equation, which can be expressed by η(T,t) = 10.16 exp (−1.72/T) exp [17.27 t exp (−5.21/T)]. After using BGAP as the adhesive to make a propellant slurry with a liquid material component of 25%, the effect of the particle size of Al powder in the solid filler component on the curing process of the slurry was studied, and the 200 nm Al powder could not be made into a slurry under this formulation. The curing kinetics equations of the slurry with Al powder particle sizes of 5 μm, 15 μm, and 29 μm under this formula were obtained by measuring the viscosity of the slurry over time at 50–70 °C. The results showed that the smaller the Al powder particle size, the lower the viscous flow activation energy of the slurry and the higher the curing reaction activation energy.

## 1. Introduction

Polyazidoglycidyl ether (GAP) is an energetic binder, and the GAP-based propellant has the advantages of low sensitivity, high energy, and low characteristic signal [1,2] and has broad development prospects [3]. However, due to the presence of bulky azide groups in the GAP side chains [4], the molecular flexibility is poor, resulting in poor mechanical properties [5], so GAP needs to be modified. There are teams in America [6,7], Europe [8,9], and Asia [5,10] researching the thermal, mechanical, and curing properties of GAP-modified materials. Compared with linear polyazide glycidyl ether (LGAP), branched polyazide glycidyl ether (BGAP) has a more rigid network structure and better mechanical properties. However, the molecular weight of BGAP viscosity is very high [11], and the complex network formed during the curing process makes the liquid viscosity even higher [12]. The flowability of the prepared propellant slurry is poor, and the viscosity is very high after adding solid fillers. The prepared propellant slurry has poor fluidity and very high viscosity after the addition of solid fillers. Guo et al. [13] found that the apparent viscosity of the solid-liquid mixture formed by the addition of solid components to the binder increased dramatically, and the viscosity of the system increased from 0.95 Pa·s to 32 Pa·s after the addition of ammonium fine perchlorate (AP), and the change of viscosity greatly affected the preparation process. Therefore, studying the rheological properties of the slurry during the preparation of BGAP propellant is helpful in selecting a more suitable propellant formulation.

The adhesive is the “skeleton” of the propellant because it bonds solid fillers that account for about 70% of the propellant with a content of about 15% [14]. The propellant components are bonded together through a curing reaction, which imparts mechanical properties to the propellant [15]. The change in viscosity during the curing process has an important impact on the curing process of the propellant slurry [16]. Lu et al. [17] predicted the curing performance of HTPB propellant by the curing performance of HTPB binder under different catalysts, and the results showed that triphenylbismuth (TPB) was an ideal catalyst for the binder system, which could increase the viscosity of the binder system from 6.37 Pa·s to 22.1 Pa·s in 4 h, and the catalytic effect on HTPB propellant slurry was also very good. RG Stecer et al. [18] judged the stiffness and relative molecular weight of the adhesive molecule by the change in viscosity of the adhesive molecule and predicted the degree of reactivity of the curing reaction through the change in modulus during the curing process.

The GAP-based propellant is a composite material formed by kneading a variety of solid-liquid components, and many experts and scholars at home and abroad have conducted many studies on the rheological properties of each component of propellant-on-propellant slurry. Lu Xinhao et al. [19] studied the curing kinetics of BGAP propellant slurry by measuring the dynamic energy storage modulus of the propellant and obtained the constitutive equation and curing reaction kinetic equation of BGAP propellant slurry. Liming He et al. [20] calculated the effect of combustion modifiers on the curing kinetics of GAP propellants by changing the dynamic storage modulus in rheology. In view of the fact that aluminum powder is the main energetic component affecting the energy of propellant, and its particle size and shape have an important impact on the rheological properties of propellant slurry, Li Baoxuan et al. [21] studied the effect of metal powder on the viscosity of solid propellant and found that adding 7% of metal powder to the formula will lead to an increase in the viscosity of the slurry, resulting in an elastic “agglomeration” phenomenon, which affects the process performance. J Zhang et al. [22] used GAP as the binder to study the effect of Al powder on the rheology of propellants, and the results showed that when the Al content was between 10% and 40% and the temperature was low, the more Al, the higher the storage modulus and complex viscosity of the slurry. At present, the chemical rheological method is used to describe the curing process of the slurry mainly by measuring the dynamic storage modulus during the curing process, and there is only a small body of literature that directly derives the curing kinetic equation through the change of apparent viscosity. In contrast, the curing parameters are directly given in the form of apparent viscosity, which can intuitively reflect the influence of the curing reaction kinetics on the rheological properties of the slurry, and the rheological equation of the fluid can be measured at the same time, which simplifies the steps of the rheological properties of the slurry. In this paper, the rheological properties of adhesive and propellants were explored by chemical rheology, and the curing kinetic equations of BGAP binder system and propellant system were obtained according to the apparent viscosity changes of three-component propellants with different particle sizes of Al powder under the same solid-liquid ratio formulation, which provided a reference for the preparation process of BGAP propellants.

## 2. Results and Discussion

### 2.1. Analysis of BGAP Rheological Properties

**Figure 1:**
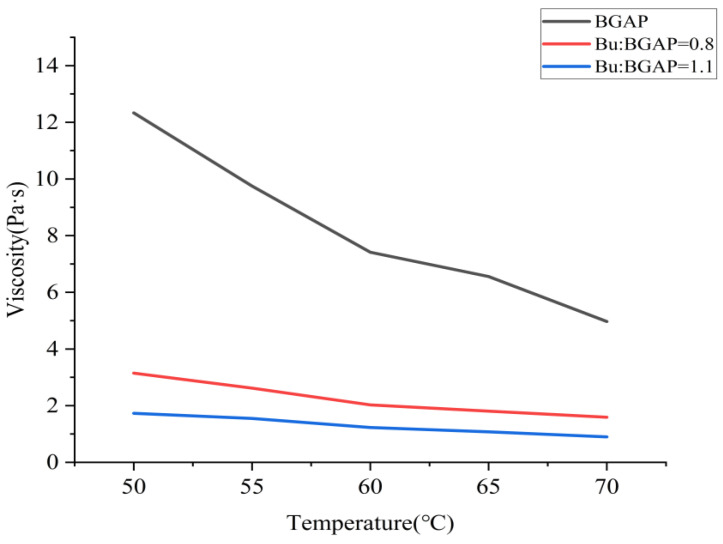
Comparison of apparent viscosity after adding plasticizers to BGAP.

**Figure 2:**
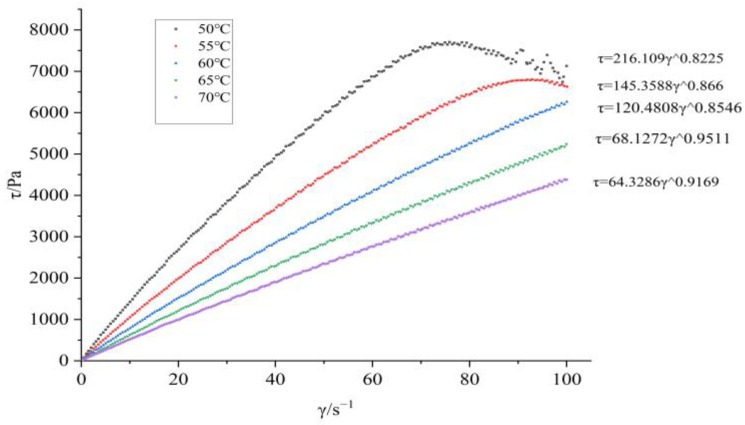
Rheological characteristic curve of BGAP.

**Figure 3:**
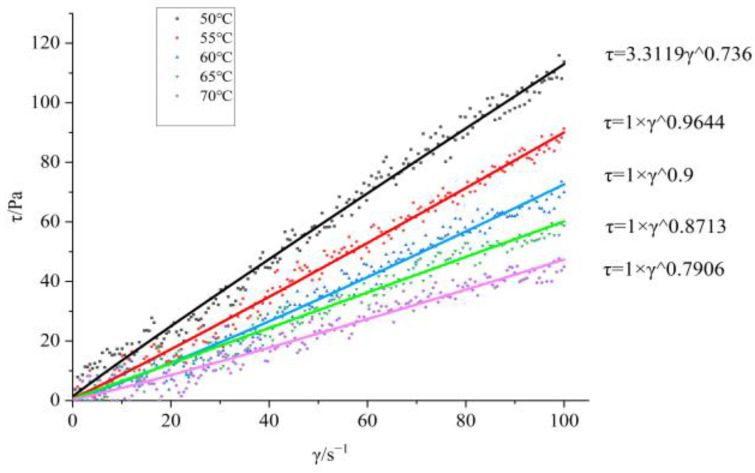
Rheological characteristic curves of solutions at different temperatures with a plasticization ratio of 1.1.

The apparent viscosity-temperature curve at a shear rate of 500 s^−1^ after adding BGAP and Bu as a plasticizer is shown in Figure 1; The shear force shear rate curves at different temperatures are shown in Figure 2 and Figure 3.

From Figure 2, it can be seen that the plasticizer Bu can significantly improve the flowability of BGAP. The larger the plasticization ratio, the better its flowability. Therefore, in practical production, adding Bu frequently to BGAP can not only improve its mechanical properties but also enhance its processability [23]. According to the change in apparent viscosity, the activation energy of viscous flow can also be obtained at different plasticization ratios.

From Figure 2 and Figure 3, it can be seen that BGAP belongs to a pseudoplastic fluid before and after adding plasticizers. The relationship between apparent viscosity, shear rate, and temperature can be described using power-law equations (Equation (1)) and Arrhenius equations (Equation (2)) [24]:

τ = K γ^n^, (1)

η = A exp (ΔE_η_/R T), (2)

In the formula, τ is the shear force, Pa; K is the viscosity coefficient, with units varying with the exponent n; γ is the shear rate, s^−1^; n is a non-Newtonian index, dimensionless; η is the Apparent viscosity, Pa·s; A is the pre-exponential factor, s^−1^; E_η_ is the activation energy of viscous flow, kJ·mol^−1^; R is the gas constant, 8.314 J·mol^−1^·K^−1^; T is the testing temperature, K.

The flow equations of the adhesive at different temperatures can be obtained from Equation (1), as shown in Table 1 and Table 2.

By taking the logarithm of Equation (2), we can obtain:

lnη = lnA + ∆E_η_/R T, (3)

**Figure 4:**
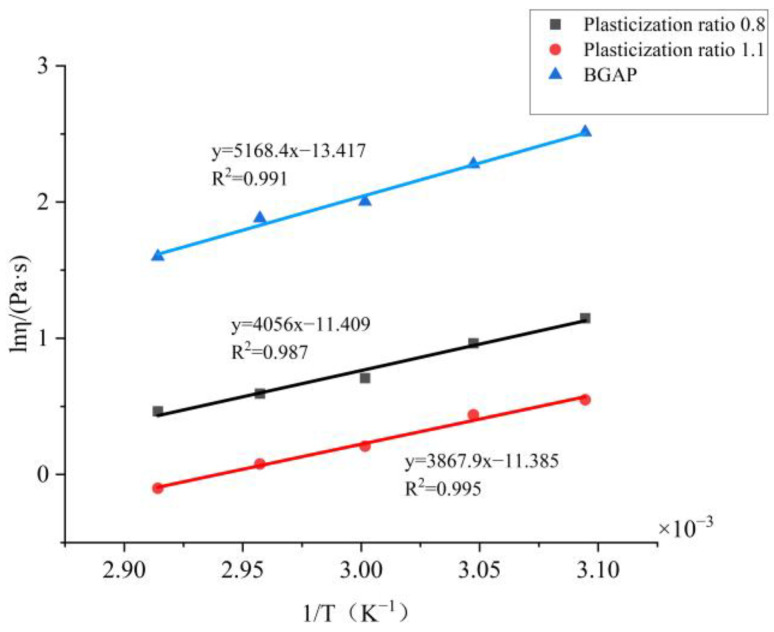
Relationship between ln η and 1/T of the BGAP mixed solution.

Based on this formula, plot BGAP and mixed solutions with plasticization ratios of 0.8 and 1.1 using ln η as the vertical axis and 1/T as the *x*-axis like Figure 4:

Based on the slope, the viscous flow activation energy E_η_ of each solution can be calculated as shown in Table 3:

The viscous flow activation energy represents the minimum energy required for a BGAP solution segment to overcome a potential barrier and transition from its original position to a nearby “hole”. It reflects both the difficulty of material flow and the temperature sensitivity of the material’s apparent viscosity changes [25]. After adding plasticizers, the solution flows more easily. According to the flow equation, it can be inferred from the viscosity coefficient K and non-Newtonian index n that the Apparent viscosity of BGAP is greatly affected by temperature, and the K value decreases significantly with increasing temperature. When Bu is added as a plasticizer, and the plasticization ratio is 1.1, the K value remains unchanged after the temperature exceeds 55 °C. Adding a plasticizer can improve the rheological properties of the adhesive, while the Apparent viscosity is more affected by temperature. According to the “lubrication theory”, plasticizers reduce the “friction” between polymers, so when the temperature increases and the molecular chain moves more vigorously, the “obstacles” to the free movement of the molecular chain are smaller.

### 2.2. Rheological Properties During the Solidification Process

**Figure 5:**
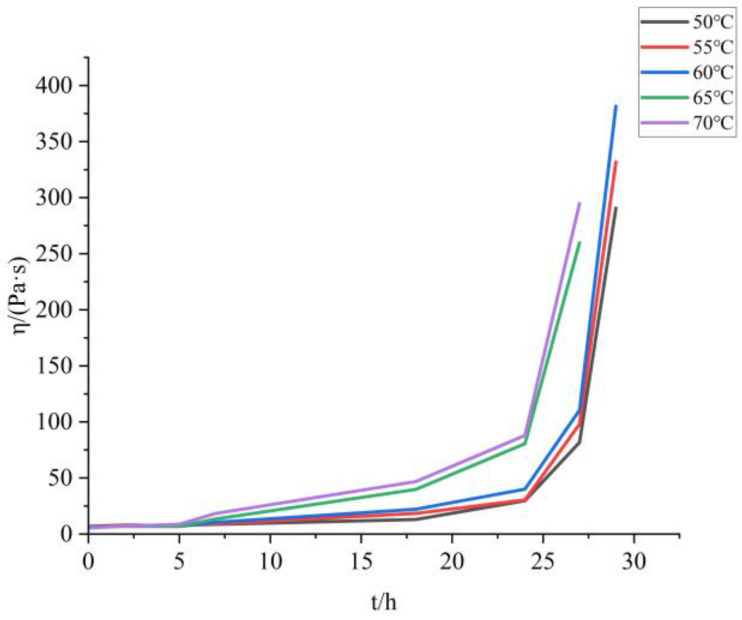
Apparent viscosity time curves of the curing process at different temperatures.

**Figure 6:**
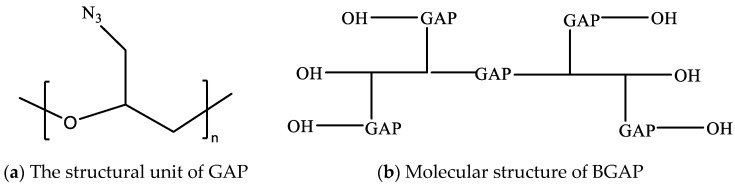
Schematic diagram of the molecular structure of BGAP.

**Figure 7:**
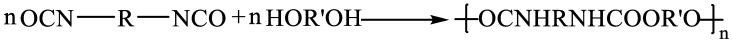
Polyurethane generation reaction.

**Figure 8:**
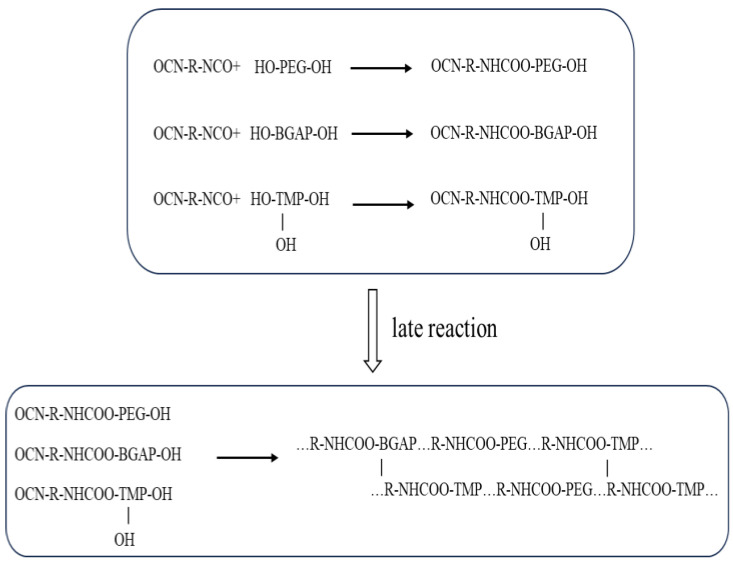
Polyurethane adhesive stepwise polymerization.

The apparent viscosity time curves of the samples with TDI and HDI added at different temperatures are shown in Figure 5:

The apparent viscosity of BAP adhesive (molecular structure shown in Figure 6) changes slowly in the early stage and even decreases with the progress of the curing reaction in the first 2 h. However, when the curing time exceeds a certain threshold, the apparent viscosity of the adhesive begins to rise sharply. This is because the reaction between hydroxyl -OH and isocyanate -NCO is a polyaddition reaction (as shown in Figure 7), which belongs to a stepwise polymerization mechanism [26]. They first form amino esters separately and then aggregate together to form macromolecules (as shown in Figure 8), similar to a condensation reaction, rather than growing from a single polymer to a high polymer, like free radical polymerization. Therefore, initially, the polymerization of each molecule did not form a cross-linked network. As the degree of reaction increased, the molecules polymerized together to form a network, resulting in a sharp increase in apparent viscosity.

Within the temperature range selected for this experiment, the temperature has two effects on the apparent viscosity of the system: on the one hand, the higher the temperature, the faster the curing reaction; On the other hand, the higher the temperature, the lower the apparent viscosity of the pseudoplastic fluid. Due to the fact that both the adhesive curing system and the propellant slurry curing process belong to pseudoplastic fluids, the same chemical rheological method can be used to calculate the curing reaction kinetics. The comprehensive effects of curing reaction and temperature can be expressed by the following equation:

η(T,t) = η_0_ exp (k t), (4)

In Equation (4), η_0_ represents the apparent viscosity of the slurry during discharge; k is the reaction rate constant, which conforms to the Arrhenius equation.

For η_0_ and k, there are:

η_0_ = η_∞_ exp (ΔE_η_/R T), (5)

k = k_∞_ exp (ΔE_k_/R T), (6)

In Equations (5) and (6), η_∞_ is the lowest apparent viscosity of the slurry when the curing degree is zero, and the temperature tends to infinity; E_η_ is the viscous flow activation energy of the solidification system; k_∞_ is the pre-exponential factor; E_k_ is the activation energy of the curing reaction; R is the gas constant; T is the testing temperature.

By substituting Equations (5) and (6) into Equation (4), the equation for the change of apparent viscosity of the drug slurry with time and temperature can be obtained [27]:

η(T,t) = η_∞_ exp (E_η_/R T) exp [k_∞_ t exp (E_k_/R T)], (7)

Taking the logarithm of Equation (7) yields the double Arrhenius formula:

lnη(T,t) = lnη_∞_ + E_η_/R T + k_∞_ t exp (E_k_/R T), (8)

It can be simplified as:

lnη(T,t) = lnA + B t,(9)

Among them:

lnA = lnη_∞_ +E_η_/R T, (10)

lnB = lnk_∞_ +E_k_/R T, (11)

**Figure 9:**
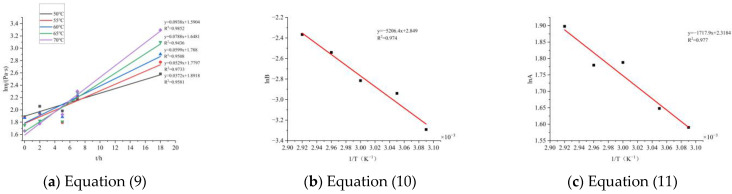
Simplified equation curve.

That is, a linear fitting can be performed on the apparent viscosity time curve to determine the curing reaction kinetics. Due to the sharp increase in apparent viscosity during the later stage of curing, it is difficult to accurately measure the apparent viscosity. Therefore, the first five points were selected for calculation, as shown in Figure 9.

After inputting the data, the parameters are shown in Table 4:

In summary, the curing kinetics equation obtained by the chemical rheological method is:

η(T,t) = 10.16 exp (−1.72/T) exp [17.27 t exp (−5.21/T)],

Taking the above equation as 333 K for 10 h and substituting it, the apparent viscosity is 9.764 Pa·s, which is very close to the actual measured value of 10.046 Pa·s. Before the apparent viscosity sharply increases, other measurement points also basically agree. Therefore, the above equation is the curing kinetics equation for the BGAP curing system measured by the rheological method.

When the curing temperature is 50 °C, it was found that the rheological equation of the adhesive still conforms to the power-law equation (as shown in Table 5) when tested at a shear rate of 1–100 s^−1^ within 100 s before 29 h. When the apparent viscosity sharply increases, R^2^ decreases and cannot be considered as a pseudoplastic fluid anymore, and the power-law equation fails. Similarly, samples with temperatures of 60 °C, 65 °C, and 70 °C can no longer be considered as pseudoplastic fluids at 27 h.

### 2.3. Rheological Properties Analysis of BGAP Propellant During Solidification Process

**Figure 10:**
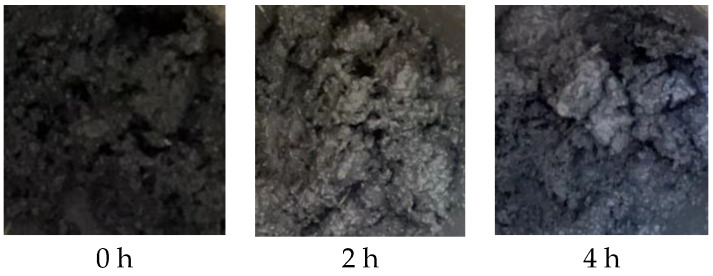
Morphology of a formula containing 200 nm Al powder after stirring and resting.

**Figure 11:**
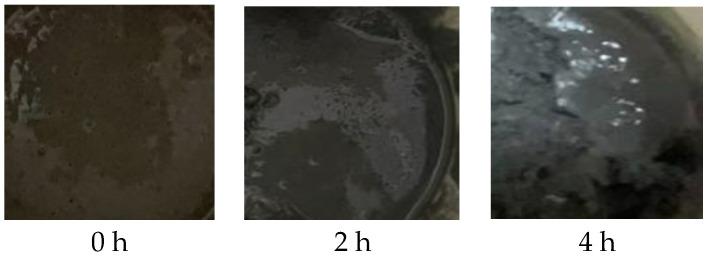
Morphology of BGAP propellant slurry after stirring and resting.

When the particle size of Al powder is 200 nm, it aggregates with other components and cannot form a flowable slurry (as shown in Figure 10). This is because the smaller the particle size of Al powder, the larger its specific surface area, and the more adhesive is required. Therefore, this formula is not suitable for using 200 nm Al powder. Micro-sized aluminum powder with a larger particle size can be used to create a flowable slurry (as shown in Figure 11). Perform apparent viscosity testing on the slurry composed of Al powder with particle sizes of 5 μm, 15 μm, and 29 μm.

**Figure 12:**
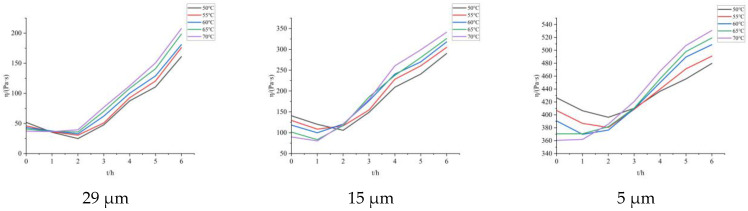
The apparent viscosity variation curve of propellants with different Al particle sizes over time.

**Figure 13:**
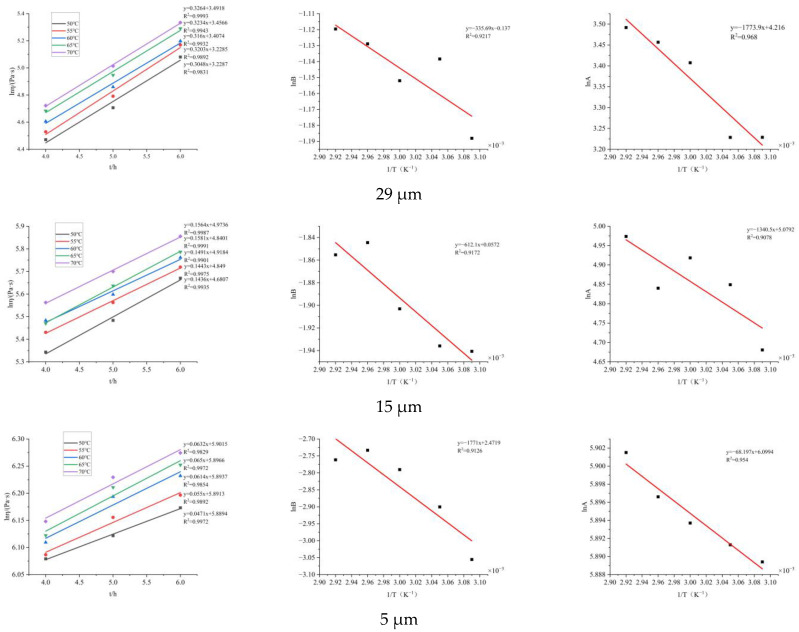
Solidification equation curves of propellant slurries with different Al particle sizes.

The apparent viscosity of propellants composed of Al powder with different particle sizes was tested every 1 h, and the results are shown in Figure 12:

Similarly, according to Figure 11, using Equations (9)–(11), the same data curve can be obtained. To predict the apparent viscosity of later solidification, the points where the apparent viscosity continues to rise are selected for graphical calculation, as shown in Figure 13. Based on this, the solidification kinetics equation of BGAP propellant represented by apparent viscosity under different Al particle sizes is calculated, as shown in Table 6:

As the particle size of Al powder decreases, its specific surface area increases, surface energy increases, and more adhesive is adsorbed. Therefore, as the particle size of Al powder increases, the viscosity of the propellant slurry increases, and the activation energy of viscous flow increases. At the same time, due to the increase in adhesive around the Al powder, the contact area between the adhesive and the curing agent increases, accelerating the curing reaction and resulting in a decrease in the activation energy of the curing reaction.

In the initial stage of curing, due to the gradual polymerization of the adhesive system, small molecular substances react first rather than directly forming a polymer network. Therefore, in the initial stage of curing, the apparent viscosity of the propellant slurry will decrease to a certain extent. To predict the subsequent changes in apparent viscosity, it is necessary to select the point where the apparent viscosity continues to increase. The selected adhesive adopts a cross-linked network curing, which is a thermosetting polymer, so the propellant slurry under this formula also has thermosetting properties. As the curing time increases, the polymer adhesive continuously undergoes cross-linking reactions in the system and the internal friction force of the slurry increases during shearing. The apparent viscosity of the propellant slurry also increases.

## 3. Materials and Methods

### 3.1. Reagents and Instruments

BGAP, The number average relative molecular weight is 20,134, and the hydroxyl value is mg KOH/g. Liming Chemical Design and Research Institute, Luoyang, China; Triphenylbismuth (TPB), Liming Chemical Design and Research Institute; 2,4-toluene diisocyanate (TDI), Sinopharm Chemical Reagent Co., Ltd., Shanghai, China; Hexamethylene diisocyanate (HDI), Aladdin Industrial Corporation, Shanghai, China; N-butylnitroethyl nitroamine (Bu), Liming Chemical Design and Research Institute; 1,1,1-Tris(hydroxymethyl)propane (TMP), Shanghai Macklin Biochemical Co., Ltd., Shanghai, China. Al powder, 5 μm, 15 μm, 29 μm, Liming Chemical Design and Research Institute; Nano Al powder, 200 nm, Xi’an Institute of Modern Chemistry, Xi’an, China; Ammonium perchlorate (AP), 100–140 mesh, Liming Chemical Research Institute; Ammonium perchlorate (AP), 6–8 μm, Liming Chemical Research Institute.

RSX rheometer, AMETEK Brookfield, Middleborough, MA, USA.

### 3.2. Experiment

The RSX rheometer was used to test the apparent viscosity of adhesives and slurries under different parameters. The method is as follows:

(1) For adhesives, shear rate scans were performed in CSR mode using a rheometer, and three types of scans were performed using a vertebral plate rotor RCTO-25-2_1111016: ① At 500 s^−1^, BGAP and BGAP with different plasticization ratios were tested for apparent viscosity at temperatures of 50 °C, 55 °C, 60 °C, 65 °C, and 70 °C. ② The temperature is 50 °C, the shear rate is set to 1–100 s^−1^, and 100 points are measured within 100 s to obtain the apparent viscosity shear rate rheological curve, which is used to derive the power-law equation in the system Conduct apparent viscosity tests on the cured adhesive at a shear rate of 1 s^−1^ at temperatures of 50 °C, 55 °C, 60 °C, 65 °C, and 70 °C to investigate its curing process.

(2) For the propellant slurry, in the CSR mode of the rheometer, a flat rotor RPTO-50_2115008 was used for scanning: apparent viscosity tests were conducted at temperatures of 50 °C, 55 °C, 60 °C, 65 °C, and 70 °C under 1 s^−1^ conditions, with tests conducted every 1 h.

#### 3.2.1. Rheological Properties Testing of BGAP Adhesive and Its Curing System

Firstly, rheological performance testing on BGAP will be performed, followed by apparent viscosity testing during the curing process. To improve the mechanical properties of the cured film, the total hydroxyl content of the TMP selected accounts for 3/5 of the total hydroxyl content [28]. The BGAP adhesive design is shown in Table 7. Before use, BGAP, BGAP/Bu, and BGAP/Bu/TMP/PEG400 mixtures are placed in a vacuum oven for dehydration. Then, according to the formula composition, each component is weighed and stirred clockwise and counterclockwise on a magnetic stirrer at 50 °C and 500 rpm for 5 min each.

#### 3.2.2. Rheological Performance Testing of BGAP Propellant

**Figure 14:**
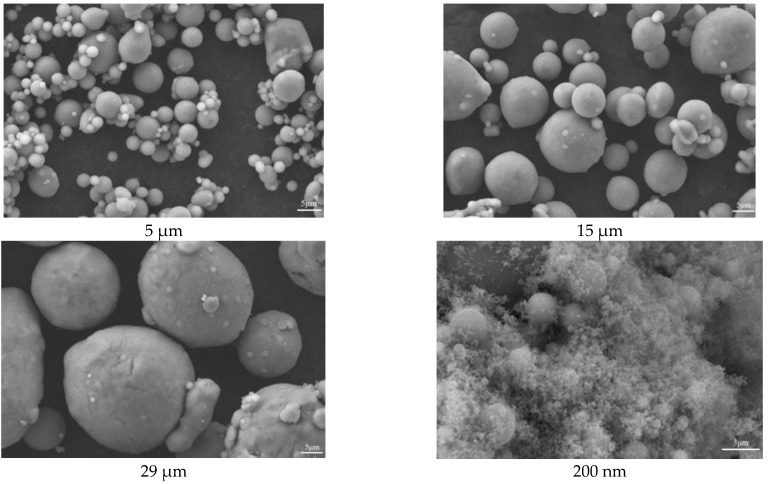
SEM images of Al powders with different particle sizes.

In this experiment, Al powder with particle sizes of 200 nm, 5 μm, 15 μm, and 29 μm was mixed with AP particles. Before the experiment, SEM tests were conducted on these solid components, and the test results are shown in Figure 14.

In the BGAP/Bu/TMP/PEG400 mixture, each component was weighed according to the formula composition and mixed evenly using a stirring blade at 50 °C, 55 °C, 60 °C, 65 °C, and 70 °C to obtain the propellant slurry for testing. The composition of the propellant slurry is shown in Table 8. The blade speed is 40 r/min, and the rotation direction is changed every 15 min. The mixing process is divided into two stages: in the first stage, Al powder is added to the mixture, which is fully coated with adhesive, and then two different particle sizes of AP are added. The particle size of 100–140 mesh is referred to as AP-1, and the particle size of 6–8 μm is referred to as AP-2. The mixture is stirred for another 10 min. In the second stage, TDI/HDI is mixed for 10 min and used as the starting point for apparent viscosity measurement.

## 4. Conclusions

(1) By measuring the apparent viscosity of BGAP adhesives with different plasticization ratios, the power-law equation and viscous flow equation were obtained, and the viscous flow activation energy of BGAP was 0.622 kJ·mol^−1^; The viscous flow activation energies of BGAP with plasticization ratios of 0.8 and 1.1 using Bu are 0.488 kJ·mol^−1^ and 0.465 kJ·mol^−1^, respectively.

(2) By measuring the apparent viscosity changes of the adhesive curing system during the curing process, the curing reaction kinetics of the adhesive curing process were studied using the double Arrhenius equation under the dual variables of temperature and reaction time. The solidification reaction kinetics equation is obtained as follows:

η(T,t) = 10.16 exp (−1.72/T) exp [17.27 t exp (−5.21/T)]

(3) By using the chemical rheological method to measure the changes in apparent viscosity of BGAP propellant slurry at different temperatures and times, the curing kinetics equations of BGAP slurry with Al powder particle sizes of 29 μm, 15 μm and 5 μm were obtained as follows:

η(T,t) = 67.76 exp (−1.77/T) exp [0.87 t exp (−0.336/T)]

η(T,t) = 160.65 exp (−1.34/T) exp[1.06 t exp (−0.612/T)]

η(T,t) = 445.59 exp (−0.68/T) exp [11.84 t exp (−1.771/T)]

According to the exponential numbers in the equation, the activation energy of viscous flow decreases with the increase of Al powder particle size, while the activation energy of the solidification reaction increases with the increase of Al powder particle size.

## Figures and Tables

**Table 1:** The rheological equation of BGAP.

Sample	T/°C	Flow Equation	R^2^
BGAP	50	τ = 216.109 γ^0.8225^	0.99897
	55	τ = 145.3588 γ^0.866^	0.99682
	60	τ = 120.4808 γ^0.8546^	0.99933
	65	τ = 68.1272 γ^0.9511^	0.99934
	70	τ = 64.3286 γ^0.9169^	0.99996

**Table 2:** Rheological equation of a mixed solution with Bu: BGAP = 1.1.

Sample	T/°C	Flow Equation	R^2^
Mixed Solution	50	τ = 3.3119 γ^0.736^	0.99814
	55	τ = 1 · γ^0.9644^	0.99510
	60	τ = 1 · γ^0.9^	0.99532
	65	τ = 1 · γ^0.8713^	0.99999
	70	τ = 1 · γ^0.7906^	0.99749

**Table 3:** Flow kinetics parameters of the BGAP mixed solution.

Sample	Viscous Flow Equation	ΔE_η_/(kJ·mol^−1^)	R^2^
BGAP	η = 1.49 · 10^−6^ exp (0.622/R T)	0.622	0.99197
Bu:BGAP = 0.8	η = 1.11 · 10^−5^ exp (0.488/R T)	0.488	0.98782
Bu:BGAP = 1.1	η = 1.14 · 10^−5^ exp (0.465/R T)	0.465	0.99533

**Table 4:** Parameter values of the BGAP curing kinetics equation.

η_∞_/(Pa·s)	E_η_/(kJ·mol^−1^)	k_∞_	E_k_/(kJ·mol^−1^)
10.16	−14.28	17.27	−43.286

**Table 5:** Rheological equation during the 50 °C curing process.

Time/h	Power Law	R^2^
0	τ = 2.1105 γ^0.794^	0.99901
2	τ = 3.838 γ^0.7148^	0.99064
5	τ = 2.3323 γ^0.8684^	0.99710
7	τ = 3.3793 γ^0.7967^	0.99158
18	τ = 9.9538 γ^0.6919^	0.99758
20	τ = 22.5585 γ^0.5707^	0.99330
24	τ = 27.4837 γ^0.6667^	0.99981
27	τ = 52.388 γ^0.5972^	0.99985
29	τ = 645.4995 γ^0.0826^	0.99366
31	τ = 1117.3159 γ^−0.0872^	0.82631

**Table 6:** The solidification equation of propellant slurry with different Al particle sizes.

Al Powder Particle Size	Curing Equation
29 μm	η(T,t) = 67.76 exp (−1.77/T) exp [0.87 t exp (−0.336/T)]
15 μm	η(T,t) = 160.65 exp (−1.34/T) exp[1.06 t exp (−0.612/T)]
5 μm	η(T,t) = 445.59 exp (−0.68/T) exp [11.84 t exp (−1.771/T)]

**Table 7:** BGAP curing system formula.

Reagent	BGAP	Bu	TPB	TMP	PEG400	TDI	HDI
Content/%	38.5	42.4	0.3	2.3	4.0	8.3	4.2

**Table 8:** Formula of BGAP propellant.

Ingredient	Liquid	Al	AP-1	AP-2
Content/%	25	15	50	10

## Data Availability

The original contributions presented in this study are included in the article. Further inquiries can be directed to the corresponding author.
